# Ixazomib–Thalidomide–Dexamethasone for induction therapy followed by Ixazomib maintenance treatment in patients with relapsed/refractory multiple myeloma

**DOI:** 10.1038/s41416-019-0581-8

**Published:** 2019-09-27

**Authors:** Heinz Ludwig, Wolfram Poenisch, Stefan Knop, Alexander Egle, Martin Schreder, Daniel Lechner, Roman Hajek, Eberhard Gunsilius, Karl Jochen Krenosz, Andreas Petzer, Katja Weisel, Dietger Niederwieser, Hermann Einsele, Wolfgang Willenbacher, Thomas Melchardt, Richard Greil, Niklas Zojer

**Affiliations:** 1 Wilhelminen Cancer Research Institute, Department of Medicine I, Center for Oncology and Hematology, Wilhelminenspital, Vienna Austria; 2 Department of Hematology, University of Leipzig, Leipzig, Germany; 3 Division of Hematology and Medical Oncology, Department of Internal Medicine II, Wuerzburg University Medical Center, Wuerzburg, Germany; 4 Department of Internal Medicine III with Haematology, Medical Oncology, Hemostaseology, Infectiology and Rheumatology, Oncologic Center, Salzburg Cancer Research Institute—Laboratory for Immunological and Molecular Cancer Research (SCRI-LIMCR), Paracelsus Medical University, Salzburg, Cancer Cluster Salzburg Austria; 5 Department of Medicine I, Center for Oncology and Hematology, Wilhelminenspital, Vienna Austria; 6 Department of Medicine I—Hematology with Stem Cell Transplantation, Hemostaseology and Medical Oncology, Ordensklinikum Linz Elisabethinen, Linz, Austria; 7 Fakultní Nemocnice Ostrava, Ostrava, Czech Republic; 8 Department of Internal Medicine V, Hematology and Oncology, Medical University Innsbruck, Innsbruck, Austria; 9 Department of Internal Medicine 3, Kepler Universitaetsklinikum GmbH, Med Campus III, Linz, Austria; 10 Department of Internal Medicine I, Ordensklinikum Linz—Barmherzige Schwestern, Linz, Austria; 11 University Medical Center of Hamburg-Eppendorf, Hamburg, Germany and University of Tuebingen, Tuebingen, Germany; 12 Oncotyrol, Center for personalized Cancer Medicine, Innsbruck, Austria

**Keywords:** Myeloma, Phase II trials

## Abstract

**Background:**

Ixazomib-revlimid-dexamethason showed significant activity in relapsed/refractory multiple myeloma (RRMM). Here, we evaluate ixazomib in combination with thalidomide and dexamethasone for induction treatment followed by ixazomib maintenance therapy in RRMM patients.

**Methods:**

Ninety patients have been included. Ixazomib–thalidomide–dexamethasone (4 mg, day 1, 8, 15; 100 mg daily; and 40 mg weekly) was scheduled for eight cycles followed by maintenance with ixazomib for one year.

**Results:**

The overall response rate was 51.1%, 23.3% achieved CR or VGPR and 10% MR resulting in a clinical benefit rate of 61.1%. In patients completing ≥2 cycles, the rates were 60.5%, 27.6% and 68.4%, respectively. Median progression-free survival (PFS) was 8.5 months in all, and 9.4 months in those completing ≥2 cycles. Response rates, PFS and overall survival (OS) were similar in patients with and without t(4;14) and/or del(17p), but PFS and OS was significantly shorter in patients with gain of 1q21. Multivariate regression analysis revealed gain of 1q21 as the most important factor associated with OS. Ixazomib maintenance resulted in an upgrade in the depth of response in 12.4% of patients. Grade 3/4 toxicities were relatively rare.

**Conclusions:**

Ixazomib–thalidomide–dexamethasone followed by ixazomib maintenance therapy is active and well tolerated in patients with RRMM.

**Trial registration number:**

NCT02410694

## Background

Proteasome inhibitors are an integral part of treatment of multiple myeloma. Until recently, bortezomib was the only available proteasome inhibitor, but lately the therapeutic armamentarium has been expanded by the introduction of carfilzomib and ixazomib.^1–7^ Therapy with bortezomib and carfilzomib requires frequent inpatient visits and trained staff for administration and surveillance. Ixazomib is a novel, effective proteasome inhibitor with a favourable toxicity profile and enables easy oral, fixed dose administration, obviating the need for frequent hospital visits.

Ixazomib has been studied in different clinical settings. Phase I/II studies showed remarkable tolerance and activity with ixazomib combinations both in relapsed/refractory^4–6^ or in newly diagnosed patients.^1,8,9^ The Tourmaline MM1 study revealed a significant prolongation of progression-free survival (PFS) with ixazomib in combination with lenalidomide–dexamethasone compared to lenalidomide–dexamethasone alone in patients with 1 to 3 prior treatment lines.^6,10^ This study indicated that ixazomib overcomes the negative impact of adverse cytogenetics.^11^ Other preliminary data show significant activity of ixazomib in combination with lenalidomide or other drugs in patients with newly diagnosed disease, both in transplant-eligible and in transplant non-eligible patients.^8,9^ Recent results of the Tourmaline MM3 study showed a significant increase in PFS with ixazomib maintenance therapy over placebo after autologous transplantation.^12^ Here we assess the activity and tolerance of ixazomib in combination with thalidomide and dexamethasone followed by ixazomib maintenance therapy in patients with relapsed/refractory multiple myeloma (RRMM).

## Patients and methods

Ninety patients with RRMM and one or more prior lines of therapy have been enrolled and started on therapy with ixazomib (4 mg on days 1, 8 and 15), thalidomide (100 mg/day), and dexamethasone (40 mg once/week). Patients aged ≥75 years received lower doses of thalidomide (50 mg/d) and dexamethasone (20 mg). Treatment was scheduled for eight 28-day cycles, followed by ixazomib maintenance therapy (4 mg, days 1, 8, 15 of a 28-day cycle, and 3 mg in patients aged ≥75 years) for 1 year. FISH analysis was performed on CD138 selected bone marrow plasma cells. The cut-off level for positivity was 10% for t(4;14), and for t(14;16), 20% for gain of 1q21, and 50% for del(17p), respectively. The cut-off level for the latter aberration was selected in accordance with findings of the IFM (Intergroupe Francophone du Myelome) group, which found this value as prognostically most relevant.^13^ High risk (HR) FISH cytogenetics were defined according to the IMWG (International Myeloma Working Group)^7^ and included patients with one or more of the following aberrations: t(4;14), t(14;16) and/or del 17p. The presence of gain of 1q21 was analysed as an additional prognostic factor.

The previously defined primary endpoint was PFS, and secondary objectives were overall response rate, overall survival (OS), impact of cytogenetic risk, of renal impairment on effectiveness, safety, myeloma frailty status^14^ and quality of life (QoL). Data on QoL will be reported separately. PFS and OS were estimated according to Kaplan-Meier^15^ and differences evaluated by log-rank test.^16^ Response rates were compared using Fisher’s Exact Test.^17^ The association of several factors with survival was tested using Cox regression analysis.^18^ Response rates are given for the PP and ITT population separately, while all other data are presented for the ITT group.

## Results

Seventy-six of the 90 patients of the intent-to-treat group (ITT) received at least two full treatment cycles and represent the per-protocol population (PP). Reasons for discontinuation were progressive disease in 9 (10.0%), toxicity in 2 (2.2%), withdrawal of patient consent in 1 (1.1%) and physician decision in 2 (2.2%) patients, respectively. A chart of the patient flow is shown as supplementary file. FISH data were available in 61 (68%) of the patients. Patient characteristics of the ITT group are shown in Table 1. The median number of prior treatment lines is 1 (range: 1–8). Forty-three (47.8%) patients have completed all eight cycles of induction therapy (median number of cycles: 6) and started ixazomib maintenance therapy, and 13 (30.2%) have completed the planned 12 cycles. The median duration of maintenance therapy was 7 months.

**Table 1:** Patient characteristics

Parameter	Patients (*n* = 90)
Age, years (range)	67.3 (44–84)
Male/female	46 (51.1)/44 (48.9)
ISS stage: I/II/III	37 (41.1)/30 (33.3) / 23 (25.5)
ECOG Status 0–1/2	86 (95.6)/4 (4.4)
*Type of MM*	
IgG/IgA	47 (52.2)/20 (22.2)
Light chain only	23 (25.6)
*Cytogenetics*	
t(4;14) and/or del(17p)	18/61 (29.5)
Gain of 1q21	32/61 (52.5)
None	22/61 (36.1)
*Prior treatment lines*	
1–2/3–4/≥5	66 (73.3)/15 (16.7)/9 (10.0)
*Prior exposure to*	
PI/IMiD/PI and IMiD	85 (94.4)/54 (60.0)/50 (55.5)
Len exposed/Len refractory	42 (46.7%)/14 (15.6%)
Thal exposed/Thal refractory	12 (13.3%)/0 (0)
Btz exposed/Btz refractory	79 (87,8%)/9 (10%)
Autologous stem cell transplantation	61 (67.8)
Months since start of 1st line TX, median (IQR)	49 (31–87)

*PI* proteasome inhibitor, *IMiD* immunomodulatory drugs, *Len* Lenalidomide, *Thal* Thalidomide, *Btz* Bortezomib

Median follow-up was 19.1 months. Response data are shown for the ITT and the PP group (Table 2). In the ITT group, partial response (PR) or better was achieved in 46 patients (51.1%), complete response (CR) in 8 (8.9%), very good partial response (VGPR) in 13 (14.4%), partial response (PR) in 25 (27.8%) and minor response (MR) in 9 (10.0%) patients, yielding a clinical benefit rate of 61.1%. In the PP group, CR, VGPR, PR, ORR and CBR was noted in 10.5%, 17.1%, 32.9%, 60.5% and 68.4%, respectively. ORR was 64.9% in the 37 IMiD naïve and 41.5% in the 53 patients with previous IMiD exposure (*p* < 0.0001).

**Table 2A:** Response rates in intent-to-treat and in the per protocol population

Parameter	ITT Population (*n* = 90) (%)	PP Population (*n* = 76) # patients (%)
CR	8 (8.9%)	8 (10.5%)
VGPR	13 (14.4%)	13 (17.1%)
PR	25 (27.8%)	25 (32.9%)
MR	9 (10.0%)	6 (7.9%)
ORR	46 (51.1%)	46 (60.5%)
CBR	55 (61.1%)	52 (68.4%)

*CR* complete response, *VGPR* very good partial response, *PR* partial response, *MR*  minimal response, *ORR*  overall response rate, *CBR* clinical benefit rate

In the 61 patients with FISH data available, 13 (21.3%) presented with t(4;14), and 6 (9.8%) with del17p. One or both features were detected in 18 patients, which comprise the high-risk group. One patient had t(14;16) and gain of 1q21. Thirty-two patients (52.5%) had gain of 1q21. HR cytogenetics and/or gain of 1q21 were detected in 39 (63.9%) patients. Response rates (≥PR) did not differ significantly between patients with conventionally defined HR features and standard-risk (SR) profile (61.1% vs. 51.2%, *p* = .578). Overall response rates were similar in patients with gain of 1q21 compared to those without gain of 1q21 (46.9% vs. 69.2%, *p* = .113), and in those with HR cytogenetics and/or gain of 1q21 compared to those without these features (46.2% vs. 68.2, *p* = .116; Table 2B).

**Table 2B:** Overall response rates by risk group

Response category	t(4;14) ± del(17p)		Significance
	Positive	Negative	
≥PR	61.1%	51.2%	*P* = 0.5778
*gain of 1q21*			
	Positive	Negative	
≥PR	46.9%	69.2%	*P* = 0.1133
*t(4;14)* *±* *del(17p)* *±* *gain of 1q21*			
	Positive	Negative	
≥PR	46.2%	68.2%	*P* = 0.1158

Of the 43 patients who had been enrolled in the ixazomib maintenance treatment phase, five (12.2%) reached a higher response category (three from VGPR to CR, and one from PR to VGPR and one from PR to CR) within 1–9 months after initiation of maintenance therapy.

**Fig. 1:**
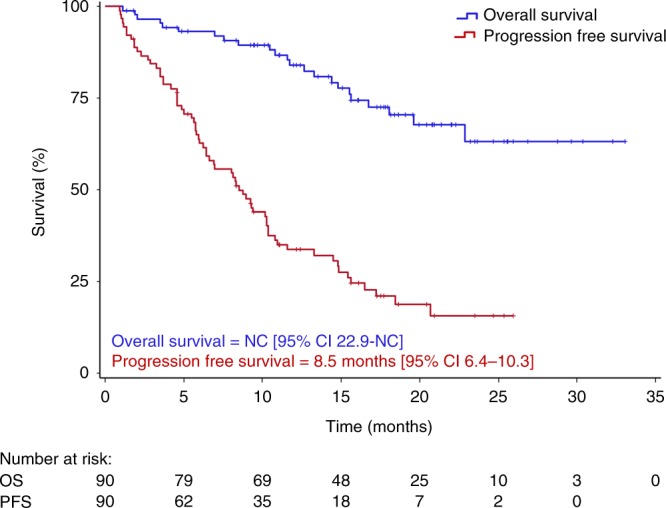
Progression-free and overall survival in the intent-to-treat group

**Fig. 2:**
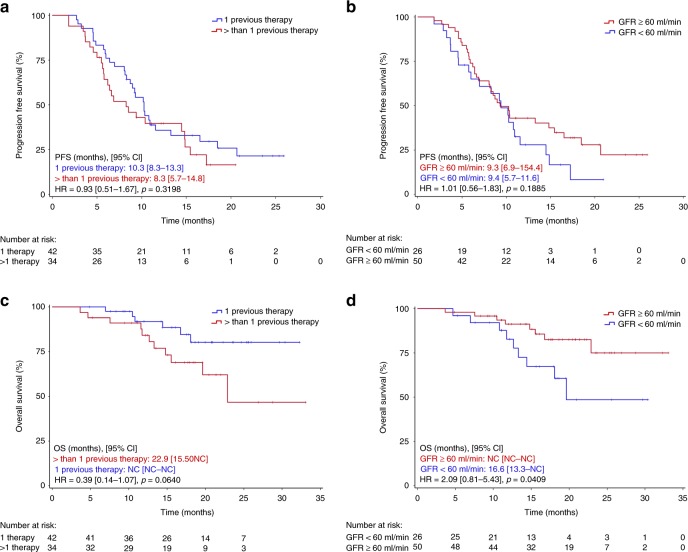
Progression-free survival (PFS) and overall survival (OS) in patients with one vs. ≥2 prior treatment lines (**a**, **c**). PFS and OS in patients with GFR < 60 ml/min and those with GFR ≥ 60 ml/min (**b**, **d**)

**Fig. 3:**
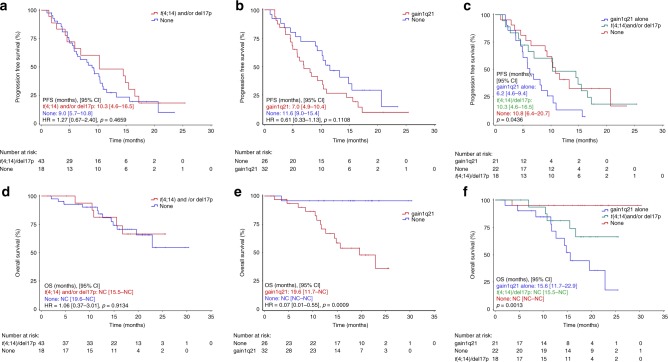
Progression-free survival (PFS) and overall survival (OS) in patients with and without high-risk (t(4;14) and/or del(17p) cytogenetics (**a**, **d**)). PFS and OS in patients with and without gain of 1q21 (**b**, **e**). PFS and OS in patients with high-risk (t(4;14) and/or del(17p) cytogenetics, with gain of 1q21 alone or neither one (**c**, **f**))

Median PFS at the time of reporting was 8.5 months in the ITT (Fig. 1) and 9.4 months in the PP group. PFS was similar in patients with ISS stage I compared to stage II and III patients (10.3 vs. 9.4 months, *p* = 0.967), in patients with one or more than one prior treatment line (10.3 vs. 8.3 months, *p* = 0.319; Fig. 2a), in patients below the age of 75 and those aged 75 years or older (10.2 vs. 9.4 months, *p* = 0.339), in fit versus unfit/or frail patients (9.2 vs. 10.9, *p* = 0.810), and in those with glomerular filtration rate (GFR) ≥ 60 ml/min or lower (9.4 vs. 9.3 months, *p* = 0.188; Fig. 2b). Similarly, no difference in PFS was noted in patients with HR and SR cytogenetics (10.3 vs. 9.0 months, *p* = 0.466; Fig. 3a) and in IMiD naïve or IMiD pre-exposed patients (10.2 vs 6.2 months, *p* = 0.026). PFS was 7.0 months in patients with, and 11.6 months in those without gain 1q21 (*p* = 0.111; Fig. 3b), and 7.0 months in patients with HR and/or gain of 1q21 versus 10.8 months in those without these features (*p* = 0.159). When PFS was compared between patients with gain of 1q21, HR cytogenetics and those with neither of those factors, a significant difference was noted for patients with gain of 1q21 (6.2 vs. 10.3 vs. 10.8 months, respectively, *p* = .044; Fig. 3c).

Median OS has not been reached, neither in the ITT nor in the PP group. Patients with stage I and II had significantly longer OS compared to stage III patients (NR vs. 14.8 months, *p* < 0.0001). Likewise, a shorter OS was noted in patients with GFR < 60 ml/min (Fig. 2d), while in those with one prior line of therapy only a tendency for longer OS was noted compared to patients with more prior lines of therapy (NR vs. 22.9 months, *p* = 0.064; Fig. 2c). Similar findings were made for fit versus unfit/frail patients (NR vs. NR months, *p* = 0.880). OS was similar in patients aged 75 years or older versus younger patients (NR vs. NR, *p* = 0.287) and in those with and without HR cytogenetics (NR vs. NR, *p* = 0.913; Fig. 3d). Patients with gain of 1q21 had significantly shorter OS compared to those without (19.6 months vs. NR, *p* = 0.0009; Fig. 3e). A similar observation was made in those patients with gain of 1q21 and/or HR cytogenetics (19.6 months vs. NR, *p* = 0.003). When OS was compared between those with isolated gain of 1q21, HR cytogenetics, and those with neither of those factors, a significant difference was noted (15.6 vs. not reached vs. not reached, *p* = 0.001; Fig. 3f).

Individual risk factors associated in univariate analysis with OS with a *p*-value < 0.2 (gain of 1q21, number of previous therapies > 1, ISS stage II-III, haemoglobin < 100 g/l, albumin < 35 g/l, GFR < 60 ml/min) were tested in a Cox multivariate regression analysis in the 60 patients with all data available. Gain of 1q21 (HR = 14.29 (95% CI, 1.69–100), *p* = 0.014), and low haemoglobin (HR = 5.00 (95% CI, 1.11–25.00), *p* = 0.034) were found to be significantly associated with shortened OS.

Adverse events are shown in Table 3. The most frequently observed adverse events were of haematologic nature. Grade 3/4 adverse events for anaemia, leukopenia, neutropenia, and thrombocytopenia were noted in 17.8%, 5.6%, 2.2%, and 7.8% of patients, respectively. The most frequent non-haematologic toxicities were infections, with grade 1/2 and grade 3/4 infections noted in 61.1% and 19.9% of patients, respectively. Severe (grade 3/4) pain was observed in 6.7%, and polyneuropathy grade 1/2 and grade 3/4 was noted in 36.7% and 4.4%, respectively. Adverse events during maintenance phase were rare and mainly grade 1 and 2.

**Table 3:** Incidence of non-haematological adverse events (stratified by grades 1–2, ≥3 during induction + maintenance phase and maintenance phase only; AEs grades 1–2 in at least 10% of patients and all ≥3 events are reported)

	Induction + Maintenance (*n* = 90)		Maintenance only (*n* = 41)	
Adverse event, *n* (%)	Grade 1/2	Grade ≥ 3	Grade 1/2	Grade ≥ 3
*Non-haematological*				
Upper respiratory infection	24 (26.7)	1 (1.1)	8 (19.5)	1 (2.4)
Lower respiratory infection	13 (14.4)	8 (8.9)	1 (2.4)	0 (0.0)
Viral infection	7 (7.8)	3 (3.3)	2 (4.9)	0 (0.0)
Urinary tract infection	1 (1.1)	2 (2.2)	0 (0.0)	1 (2.4)
Infection unspecified	10 (11.1)	2 (2.2)	2 (4.9)	0 (0.0)
Varicella zoster virus inf.	0 (0.0)	1 (1.1)	0 (0.0)	0 (0.0)
Sepsis	0 (0.0)	1 (1.1)	0 (0.0)	0 (0.0)
Polyneuropathy	33 (36.7)	4 (4.4)	7 (17.1)	1 (2.4)
Fatigue	33 (36.7)	2 (2.2)	4 (9.8)	0 (0.0)
Constipation	26 (28.8)	1 (1.1)	1 (2.4)	0 (0.0)
Diarrhoea	8 (8.9)	3 (3.3)	1 (2.4)	1 (2.4)
Nausea	16 (17.8)	0 (0.0)	3 (7.3)	0 (0.0)
Oedema	26 (28.9)	0 (0.0)	4 (9.8)	0 (0.0)
Pain	22 (24.4)	6 (6.7)	9 (22.0)	0 (0.0)
Renal disorders	2 (2.2)	2 (2.2)	0 (0.0)	0 (0.0)
Cardiac disorders	4 (4.4)	2 (2.2)	0 (0.0)	1 (2.4)
Secondary malignancies	1 (1.1)	2 (2.2)	0 (0.0)	0 (0.0)
*Haematological*				
Anaemia	66 (73.3)	16 (17.8)	34 (82.9)	4 (9.6)
Leukopenia	46 (51.1)	5 (5.6)	28 (68.3)	1 (2.4)
Neutropenia	35 (38.9)	2 (2.2)	17 (41.5)	0 (0.0)
Thrombocytopenia	36 (40)	7 (7.8)	17 (41.5)	3 (7.3)

## Discussion

The all-oral ixazomib–thalidomide–dexamethasone combination followed by ixazomib maintenance therapy revealed an overall response rate of 51.1% and clinical benefit rate of 61.1% in the ITT, and of 60.5 and 68.4%, respectively, in the PP population (Table 2A). Five of the 41 (12.2%) patients starting ixazomib maintenance therapy experienced a deepening of their responses after 1–9 months from start; at the time of data cut-off, 10 patients were still on therapy. The observed response rates compares well with the 56.3%, and 68.3% overall responses observed with the same drug combination within the Myeloma XII study (Accord trial) in younger patients with biochemical and clinical first relapse after autologous transplantation, respectively.^19^ Slightly lower response rates (48%) were reported with ixazomib–pomalidomide in a small series of lenalidomide refractory patients.^20^ Similar efficacy data with an overall response rate of 48% were obtained in a phase II study employing weekly cyclophosphamide-dexamethasone in combination with ixazomib in patients with relapsed/refractory multiple myeloma after 1–3 prior lines of therapy,^21^ but median PFS was slightly longer (14.2 months).

The PFS results observed here (Figs. 1 and 3a–c) need to be interpreted in the context that the median time from start of first line therapy to inclusion in this study was 49 months and that 18 (20%) of our patients were more heavily pre-treated (4–8 prior treatment lines). The high number of patients with adverse cytogenetics (either HR or gain of 1q21, *n* = 39, 63.9%) underlines the fact that several of them had advanced disease, and the majority had been pre-treated with novel agents. Almost all (94.4%) had previously been exposed to PI, 60% to IMiDs, and 55.5% to both PIs and IMiDs.

The median PFS of 8.5 months in the ITT and of 9.4 months of PP groups is comparable to findings obtained in several other phase II studies including similar patient populations. Although cross trial comparisons should be interpreted with caution, recent phase I/II studies utilising pomalidomide–cyclophosphamide-dexamethasone yielded a response rate of 65% and a PFS of 9.5 months.^22^ Similar findings with a response rate of 60% and a PFS of 8.8 months were noted in more heavily pre-treated relapsed/refractory myeloma with daratumumab in combination with pomalidomide and dexamethasone.^23^ In patients refractory to lenalidomide, a PFS of 4.3 and 10.3 months, respectively, was noted in those treated with pomalidomide–dexamethasone and in the group receiving elotuzumab in combination with the pomalidomide–dexamethasone backbone.^24^ Some of the recent large phase III trials conducted for applying for approval reported similar PFS data in the control arms, but significantly longer PFS in the experimental arms.^25,26^ It should be noted that in this academic trial the screening phase was very short (median 7 days), indicating less stringent patient selection than in studies aiming at drug approval (screening phase usually 3 weeks) and suggesting that the present study contains a broader representation of patients that may be more similar to those in clinical everyday practice with usually less favourable prognosis.

The treatment regimen was equally effective in terms of PFS data when analysing subgroups according to ISS stage (I + II vs. III), age (<75 vs. ≥75 years), fitness (fit or unfit vs. frail), GFR (<60 ml/min vs. ≥60 ml/min; Fig. 2b), number of previous treatment lines (Fig. 2a), and HR cytogenetics (Fig. 3a–c). PFS was 7.0 months in patients with gain of 1q21 and 11.6 months in those without this risk factor (HR = 0.61 (95% CI, 0.33–1.13), *p* = 0.111). Interestingly, when PFS was compared between patients with gain of 1q21, those with HR cytogenetics and those with neither gain of 1q21 nor HR features, respectively, PFS was found to be significantly shorter in patients with gain of 1q21, while between the two other groups no difference was observed.

The similar efficacy of this ixazomib-based regimen in patients with and without HR cytogenetics is compatible with the results obtained in the TOURMALINE-MM1 study, where the negative impact of HR cytogenetics defined as t(4;14) and/or del(17p) was overcome with ixazomib in combination with lenalidomide–dexamethasone.^10^ Our data show similar activity of the ixazomib–thalidomide–dexamethasone regimen in the cytogenetic HR and in the SR group in terms of response rate, PFS and of OS. However, in patients with gain 1q21, either as single risk factor, or with or without t(4;14) and/or del(17p) a significantly shorter OS was noted (Fig. 3e, f).

Similar observations have been reported for the TOURMALINE-MM1 study.^10,11^ Patients with gain of 1q21 had a lower improvement in PFS compared to Rd (HR = 0.781), while in patients with del(17p) (HR = 0.596) or t(4;14) (HR = 0.645) a greater PFS benefit was noted.^11^ The median PFS did not differ between HR and SR (21.4 and 20.6 months, respectively) patients, but noteworthy, was 6 months shorter in patients with gain of 1q21.

Cox regression analysis revealed gain of 1q21 as most important prognostic parameter associated with shortened overall survival. This finding and data published by others^11,27,28^ suggest that gain of 1q21 should be included in the HR category in patients exposed to ixazomib plus thalidomide-dexamethasone, and probably in all other studies investigating the impact of cytogenetics.^26^ The frequency of 1q21 gains increases with progressing disease.^29^ Several genes located in this region, such as PSMD4,^30^ CKS1B^31^ MUC1, MCL, ILF2 and others have been associated with increased myeloma progression and likely account for the poorer outcome of patients with gain within this gene region. In this study with a limited number of patients with gain of 1q21, we found no differences in the PFS and OS between patients with three, or four or more copies of 1q21. It remains unresolved whether the addition of thalidomide contributed to the reduced survival in patients with gain of 1q21. Previous studies with thalidomide maintenance therapy showed significantly shortened OS in elderly patients with HR cytogenetics.^32^ Similar findings were reported from Poland in a cohort of patients receiving thalidomide-based first line therapy.^28^ In this patient cohort, gain of 1q21 was the most important cytogenetic factor associated with shortened PFS and was also found to closely correlate with poor OS. These observations are of clinical relevance for most parts of the globe, where thalidomide still is the only available IMiD for treatment of myeloma patients.^33^ Expectedly, a tendency for shorter OS was noted in patients with two or more prior treatment lines (Fig. 2c), while in those with impaired renal function (GFR < 60 ml/min) a significantly shorter OS was observed (Fig. 2d). As increasingly more drugs and treatment strategies have shown efficacy in RRMM patients,^34^ selection of the IxaThalDex regimen depends, among other factors, on local availability of drugs. As access to lenalidomide is still not possible for 90% of the global population of multiple myeloma patients, combining thalidomide with an oral proteasome inhibitor is a valuable option for RRMM patients. Induction therapy with IxaThalDex yielded remarkable efficacy (ORR: 81%, ≥VGPR 47%) and was very well tolerated in elderly newly diagnosed patients.^7^ Hence, it may also be considered for first line therapy in patients opting for an oral combination regimen. Among the limitations of our study, the lack of a randomised comparison with other established regimens, and missing FISH data in 32% of patients should be considered.

The treatment regimen was well tolerated, with infections being the most frequent non-haematologic adverse events (Table 3). Most of the infections were grade 1/2, with severe infections (grade 3/4) noted in only 20% including sepsis in one patient, who recovered from this complication. Remarkably, peripheral neuropathy grade 1/2 was noted in 32% of patients already at baseline. Worsening to grade 3/4 was observed in 5% of patients despite thalidomide exposure for a median of 6 months. Even though thalidomide has been given in lower doses (50 mg daily to patients aged ≥ 75 years, and 100 mg daily to the younger patients) than in many previous studies, it is tempting to speculate whether ixazomib alleviates thalidomide-induced neuroinflammation by reducing NF-κB signalling, which has been implicated in inducing neuropathic pain.^34^

In summary, the results show clinically valuable activity of the all oral ixazomib–thalidomide–dexamethasone followed by ixazomib maintenance in patients with and without HR cytogenetics. Treatment outcome was less favourable in patients with gain of 1q21 either as single abnormality or in combination with other HR cytogenetic lesions. Ixazomib maintenance therapy resulted in an upgrade in response category in 12.2% of patients. Patients tolerated induction therapy and maintenance therapy remarkably well with a relatively low incidence of grade 3/4 neuropathy.

## Supplementary information

Flow chart

List of ethics comittees of all countries

## Data Availability

The data are available for all study authors. The datasets used and analysed during the current study are available from the corresponding author on reasonable request.
