# Supplementary figures and images for: Ixazomib–Thalidomide–Dexamethasone for induction therapy followed by Ixazomib maintenance treatment in patients with relapsed/refractory multiple myeloma
Source: Br J Cancer. 2019 Sep 27;121(9):751–7. doi: 10.1038/s41416-019-0581-8 (PMC6889132; doi:10.1038/s41416-019-0581-8)

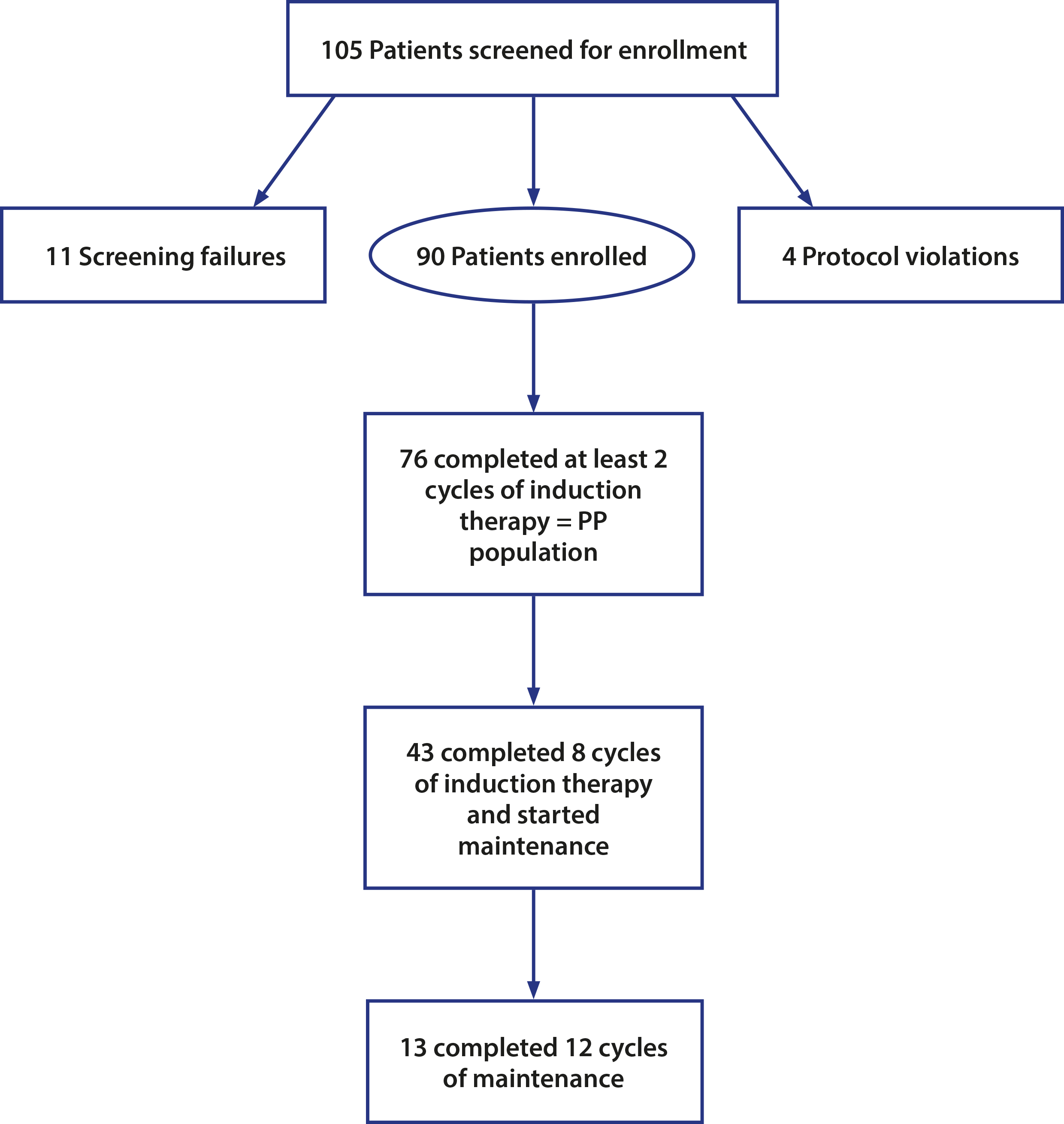

Supplement: Supplementary file 1 — Flow chart [file 41416_2019_581_MOESM1_ESM.tif]
