# Supplementary material for: Ixazomib–Thalidomide–Dexamethasone for induction therapy followed by Ixazomib maintenance treatment in patients with relapsed/refractory multiple myeloma
Source: Br J Cancer. 2019 Sep 27;121(9):751–7. doi: 10.1038/s41416-019-0581-8 (PMC6889132; doi:10.1038/s41416-019-0581-8)
Supplement: Supplementary file 2 — List of ethics comittees of all countries [file 41416_2019_581_MOESM2_ESM.docx]

**List of ethics committee’s:**

**Austria:**

| **Leading ethics committee** |
| --- |
| **Ethikkommission der Stadt Wien**  **Reference number: 14-249-1214** |

| **local ethics committees** |
| --- |
| **Ethikkommission der Medizinischen Universität Innsbruck** |
| **Ethikkommission des Landes Oberösterreich** |
| **Ethikkommission für das Bundesland Salzburg** |
| **Ethikkommission der Medizinischen Universität Wien** |
| **Ethikkommission Ordensklinikum Linz Barmherzigen Schwestern** |
| **Ethikkommission Krankenhaus der Barmherzigen Brüder Wien** |
| **Ethikkommission des Landes Vorarlberg** |
| **Ethikkommission der Medizinischen Universität Graz** |

**Germany**:

| **Leading ethics committee** |
| --- |
| **Ethik-Kommission an der Medizinischen Fakultät der Universität Leipzig**  **Reference number: 254/15-ff** |

| **local ethics committees** |
| --- |
| **Ethik-Kommission der Medizinischen Fakultät der Eberhard-Karls-Universität und Universitätsklinikum Tübingen** |
| **Ethik-Kommission der Medizinischen Fakultät der Universität Würzburg/ Institut für Pharmakologie und Toxikologie** |

**Czechia**:

| **Leading ethics committee** |
| --- |
| **Eticka komise Fakultni nemocnice Ostrava**  **Reference number: 196/2016** |

| **local ethics committee** |
| --- |
| **Etická komise FN Brno** |
